# Undiagnosed Sturge-Weber Syndrome as a Differential Diagnosis of Seizures and Deep Cerebral Venous System Dilation: A Case Report

**DOI:** 10.7759/cureus.19111

**Published:** 2021-10-29

**Authors:** Raphael Bertani, Bruno Garret, Caio M Perret, Sávio Batista, Stefan W Koester, Rodrigo Azeredo, Tamires Guimarães Cavalcante Carlos de Carvalho, José A Almeida

**Affiliations:** 1 Neurosurgery, Hospital Municipal Miguel Couto, Rio de Janeiro, BRA; 2 Neuroscience, Federal University of Rio de Janeiro, Rio de Janeiro, BRA; 3 Neurosciences, Federal University of Rio de Janeiro, Rio de Janeiro, BRA; 4 Medicine, Vanderbilt University School of Medicine, Nashville, USA; 5 Surgery, Unversidade Nove de Julho - UNINOVE, São Paulo, BRA

**Keywords:** sturge-weber syndrome, encephalotrigeminal angiomatosis, late-onset seizures, cerebral venous system dilation, undiagnosed sturge-weber syndrome

## Abstract

Sturge-Weber syndrome (SWS) is a capillary-venous malformation affecting the brain, the eye, and the adjacent trigeminal dermatomes of the skin. This illness is usually diagnosed during the first years of life. If left undiagnosed (and consequently untreated), the condition could develop into severe refractory seizures, ischemic strokes, visual loss, and early cognitive impairment.

We report a case of a 23-year-old female patient with a port-wine facial stain, presenting her first convulsive episode in adulthood, associated with a moderate dilation of the deep venous system in the angiography, which raised the diagnostic of Sturge-Weber syndrome.

## Introduction

Encephalotrigeminal angiomatosis, also known as Sturge-Weber syndrome (SWS), is a rare sporadic congenital disease, characterized by capillary-venous malformations, affecting the brain, the eye, and the adjacent trigeminal dermatomes of the skin, with an estimated incidence as low as 1 out of 20,000 to 50,000 live births [1,2]. However, it has been suggested that a portion of patients may be left undiagnosed [2]. It originates from a malformation of an embryonic vascular plexus within the cephalic mesenchyme, approximately between 5 to 8 weeks of pregnancy [3]. This disease is usually related to a *GNAQ2* gene mutation, which leads to unilateral leptomeningeal, choroidal and adjacent neural tissue vascular malformations (most occipital and posterior parietal lobe), as well as ipsilateral skin hemangiomas (usually affecting the upper face in a distribution of the ophthalmic division of the trigeminal nerve) [4,5,6]. It is essential to observe that most children with facial cutaneous vascular malformation do not have SWS, so the clinical presentation must include the two other components of the syndrome [7].

Natural history entails progressive neurological impairment unless early treatment is established [6]. Clinically, most children reach age-appropriate development in the first few months of life, but approximately half of all patients with SWS will eventually present with delayed development [8,9]. Deficits can be acquired progressively over time or acutely due to stroke-like episodes associated with seizures and migraines [8]. Most adults with SWS brain involvement have some degree of neurological deficit, such as intractable epilepsy, permanent weakness, hemiatrophy, visual field cuts, glaucoma, and mental retardation [1,2,9,10].

Management of seizures and stroke-like episodes is the key to preventing the progression of neurological injury in SWS since adjacent brain parenchyma is likely suffering from hypoxia due to microvasculature dysfunction, causing progressive brain injury. Therefore, early recognition and aggressive management of seizures are mandatory [11].

We report a case of a 23-year-old female patient sustaining late-onset seizures and presenting a moderate dilation of the deep venous system in the CT angiography scan. The further cerebral angiographic study showed the angiomatous brain-staining along the dilated portions of the deep venous system, suggesting the SWS diagnosis.

## Case presentation

**Figure 1:**
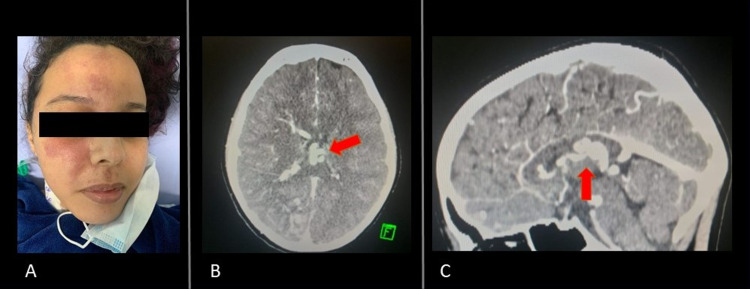
A picture of the patient showing a facial port-wine stain (A). CT angiography scan in axial (B) and sagittal planes (C) showing deep venous system dilation (red arrows).

A 23-year old female patient was referred to the neurosurgery department due to a history of seizures and transitory left hemiparesis. Her seizures began six months prior, and the hemiparesis was resolved upon admission, having lasted 48 hours. The patient was otherwise healthy, with no remarkable history of neurological disorders in the family. The neurological exam was unremarkable. A right hemifacial frontal and maxillary angiomatous skin lesion was found ectoscopically, to which the patient reported having been previously evaluated, receiving the diagnosis of “a benign birthmark” (Figure 1A).

**Figure 2:**
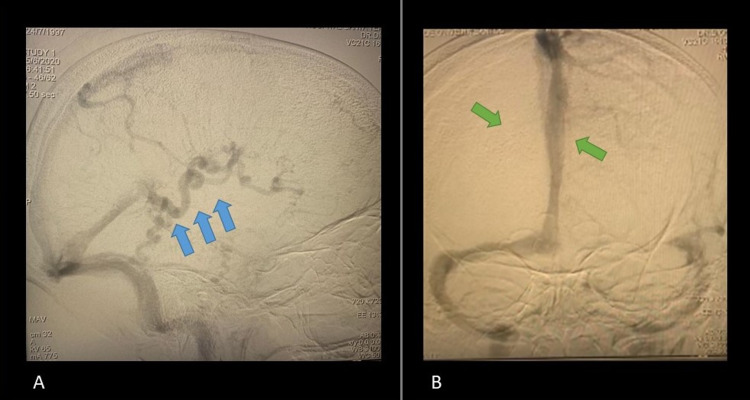
Lateral (A) and frontal (B) cerebral angiography. The lateral view shows an enlargement of the internal cerebral, Rosenthal’s basal, and Galen’s veins (blue arrows). Associated reduced flow and diameter of the right hemispheric cortical veins with straight sinus draining (green arrows) is shown in the frontal view.

Initial brain angiographic CT showed dilation of the deep venous system (Figure 1B, 1C). Diagnostic cerebral angiography was performed through the right femoral artery with a “Headhunter 1” catheter (HH1), which showed the enlargement of the right deep brain venous system (internal cerebral, Rosenthal’s basal, and Galen’s veins), draining to the straight sinus. Associated reduced flow and diameter of the right hemispheric cortical veins were also seen, suggesting the diagnosis of SWS (Figure 2).

Antiepileptic drugs were optimized, and the patient was referred to dermatological and ophthalmological evaluations to investigate and treat the other components of the syndrome.

## Discussion

The appearance of a facial cutaneous vascular malformation covering a portion of the upper or the lower eyelids in newborns, since port-wine stains affecting the entire V1 distribution (ophthalmic division of the trigeminal nerve), are strongly predictive of underlying neurological or ocular disorders, what was seen in 80.6% of the cases by Waelchli et al. [12]. Furthermore, a classical imaging exam can be performed to screen a possible intracranial leptomeningeal angiomatosis [13]. However, even in non-investigated cases, it is known that Sturge-Weber syndrome can present typically with childhood seizure, with an estimated incidence of 75% to 95% of children developing it by five years of age [9]. Therefore, it would be expected the diagnosis to be made in the first years of life based on classical imaging or presentation with seizures.

Seizures beginning in adult life require special attention since they are usually due to an identifiable cause [14]. These are mainly due to trauma, central nervous system infections, tumors, cerebrovascular accidents, metabolic disorders, and drugs [14]. SWS clinical presentation is a possible diagnosis in patients with seizures in adulthood without a previous history of seizures. In these nonclassical presentations, a diagnosis can be achieved with angiography.

Low-flow angiomatosis involving the leptomeninges accompanied by poor superficial cortical venous drainage and enlarged regional transmedullary veins developed as alternate venous drainage pathways are typical imaging findings associated with SWS [15]. Associated with the clinical scenario presented, transient focal deficits are also a common feature of SWS, with the most common manifestation being transient episodes of hemiparesis or visual field defects due to a self-limited thrombosis or to status epilepticus, which is frequently associated with prolonged weakness on one side of the body or new-onset visual field deficit (lasting several days, weeks, or months) [5,16].

According to the definition that the majority of patients with SWS presents symptoms while in childhood, marked by severe and progressive neurological deficits, it is expected that a child born with a facial SWS who has a normal neurological examination, no history of seizures, and a normal MRI with contrast after the age of 1 year probably does not have brain involvement [17].

Regarding genetics, both SWS and non-syndromic port-wine stains are thought to originate due to altered fetal development caused by a somatic mutation of the *GNAQ* gene [4]. Specifically for SWS, a study has shown that 100% of patients are positive for a c.548G→A (guanine to adenosine) mutation in the skin with the port-wine stain and 88% are positive for the same mutation in either the port-wine-stained skin or brain tissue samples.

## Conclusions

We report the case of a patient presenting with seizures in adulthood, absence of a previous diagnosis of epilepsy, a normal development, and was unexpectedly diagnosed with SWS. Diagnostic angiography showed dilation of the deep venous system, for which no treatment was warranted. She was referred to ophthalmology and dermatology for further investigation and treatment. Genetic analysis was unavailable.

Therefore, this case reinforces the importance of early clinical diagnosis and/or suspicion of Sturge-Weber syndrome, as well as it should be considered as a possible differential diagnosis when a stroke-like episode or first seizure episode is seen in early adulthood when considering a patient with clinical stigma such as a facial port-wine stain.
